# Examination on the risk factors of cholangiocarcinoma: A Mendelian randomization study

**DOI:** 10.3389/fphar.2022.900424

**Published:** 2022-08-26

**Authors:** Lanlan Chen, Zhongqi Fan, Xiaodong Sun, Wei Qiu, Wentao Mu, Kaiyuan Chai, Yannan Cao, Guangyi Wang, Guoyue Lv

**Affiliations:** Department of Hepatobiliary and Pancreatic Surgery, The First Hospital of Jilin University, Changchun, Jilin, China

**Keywords:** cholangiocarcinoma, cholelithiasis, NAFLD, risk factors, Mendelian randomization

## Abstract

**Background:** Several risk factors have been identified for CCA, however, whether such associations were causal remains unknown.

**Methods:** Mendelian randomization (MR) has been applied to examine the causal relationship between 26 putative risk factors and CCA. The genetic variants for each risk factor were extracted from their corresponding genome-wide association study (GWAS) if they reached the genome-wide significance (*p*-value < 5 × 10^−8^). The genetic associations with CCA were obtained from the publicly available GWAS with the largest sample size. Mainly, inverse-variance weighted (IVW) has been adopted to estimate the causal effect on CCA. Both multivariable and mediation MR analyses were carried out to detect independent factors.

**Results:** Three putative risk factors can causally elevate the risk of CCA after FDR correction, including liver fat content (LFC), non-alcoholic fatty liver disease (NAFLD), and cholelithiasis. The odds of CCA would increase per 1-SD increase in the liver fat content (LFC) (OR = 2.12 [1.66, 2.71]) and logOR of NAFLD. The genetic liability to cholelithiasis would increase the risk of CCA as well (OR = 2.17 [1.47, 3.20]). They were still significant in other methods. The multivariable MR analysis indicated that genetically-elevated LFC should increase the risk of CCA independently of cholelithiasis (OR = 1.88 [1.39, 2.55]). In the mediation MR analysis, the indirect effect was not significant when treating cholelithiasis as the mediator (indirect OR = 0.95 [0.85, 1.07]).

**Conclusion:** This MR study identified that gallstone and liver fat accumulation are two independent risk factors of CCA, suggesting two modifiable ways of preventing CCA.

## Introduction

Cholangiocarcinoma (CCA) is an aggressive malignancy of the bile duct and its incidence and mortality rates are increasing globally (Banales et al., 2020). CCAs are often diagnosed in the advanced stages as they usually display few symptoms in the early stage, suggesting a poor prognosis (Banales et al., 2016). The mainstream therapeutics of unresectable or metastatic CCAs are palliative chemotherapies, while the median overall survival (OS) is far from satisfactory (11.7 months) (Banales et al., 2016; Abou-Alfa et al., 2020). Even with only the approved CCA-targeted drugs, pemigatinib-targeting fibroblast growth factor receptor (FGFR) fusion and ivosidenib-targeting isocitrate dehydrogenase (IDH)-1 mutation, the median OS still represents less than 22 months in advanced CCA patients (Abou-Alfa et al., 2020; Zhu et al., 2021). Thus, it is important to identify modifiable risk factors of CCA. Recently, the consensus statement of CCA summarized its potential risk factors (Banales et al., 2020), and the majority of them are derived from a meta-analysis that systematically scrutinized 13 risk factors of CCA (Clements et al., 2020). These risk factors include primary sclerosing cholangitis (PSC), cysts and stones in the bile duct, cirrhosis, liver fluke, chronic hepatitis B or C viruses, non-alcoholic fatty liver disease (NAFLD), chronic pancreatitis, type 2 diabetes mellitus (T2DM), obesity, hypertension, inflammatory bowel disease (IBD), hemochromatosis, smoking, and alcohol consumption. It should be noted that most of these risk factors were determined based on case-control studies and whether such associations are causal are largely unknown.

Classical case-control studies based on observational data can only delineate the associations between the risk factors and targeted outcomes while failing to judge whether such associations are causal due to the existence of confounders (D'Onofrio et al., 2020). Mendelian randomization (MR) is a rising method of causal inference in genetic epidemiology using genetic variants as instrumental variables (IV) and has made substantial contributions to medical research (Emdin et al., 2017). For example, Voight et al. ruled out the protective effect of HDL cholesterol on myocardial infarction, correcting the mindset that HDL-C is a “good lipid” (Voight et al., 2012). As germline genetic variants are randomly allocated at conception, they should be free of potential confounders and MR can be a proxy for randomized control trials (RCT) (Smith and Ebrahim, 2003). Furthermore, MR can usually be performed based on observational data and is a cost-effective and time-saving method compared to RCT.

Until now, no studies have examined the causal relationship between the risk factors and CCA yet and it can be ascribed to the lack of a genome-wide association study (GWAS) on CCA. Several MR studies examined the causal risk factors of gallbladder cancer (GBC) where they identified that gallstones should be a common risk factor of GBC in Chileans (Barahona Ponce et al., 2021), Chinese (Pang et al., 2021), and Indians (Mhatre et al., 2021). However, the causal effect of the body mass index (BMI) on GBC was inconsistent as there was a positive causal effect in Chileans (Barahona Ponce et al., 2021) while it attenuated to zero in Europeans (Fang et al., 2021), suggesting that ethnical specificity should be taken into account. The germline genetic variants of CCA have been established in the genomic feature analysis of Japanese and Italian populations (Wardell et al., 2018) and it is rational to examine the causal effects of putative risk factors on it. Here, we hope to leverage the published GWAS summary statistics to identify the causal risk factors of CCA using the MR method.

## Materials and methods

### Data source description

The GWAS summary statistics of predefined risk factors were obtained from several large GWAS consortia and can be divided into six categories, including hepatobiliary diseases and their associated indices, obesity-related traits, diabetes and its associated traits, blood lipids, blood pressure, and smoking and drinking. Since their GWAS summary statistics are unavailable, the cystic diseases of the bile duct were not included as the exposures.

The hepatobiliary diseases consisted of cholelithiasis, chronic pancreatitis, non-alcoholic fatty liver disease (NAFLD), primary biliary cholangitis (PBC), primary sclerosing cholangitis (PSC), and liver cirrhosis. As an essential index for NAFLD, liver fat content was also incorporated into this modality. The cases of cholelithiasis from Iceland were denoted according to the International Classification of Disease (ICD) codes (ICD-10 K80 and ICD-9 574) and those from the United Kingdom Biobank (UKB) were determined based on the self-reported non-cancer illness (Data-Field 20002) and ICD diagnoses (ICD-9 code 574 or ICD-10 code K80) (Ferkingstad et al., 2018). The chronic pancreatitis cases were diagnosed by the ICD-10 code K11 and the cases of liver cirrhosis were selected using a broad definition as previously described by Emdin et al. (2020). All NAFLD cases were confirmed in both CT-proven and biopsy-proven steatoses (Speliotes et al., 2011). Considering that liver fat content (LFC) is an essential indicator for liver steatosis, the genetic proxies for LFC were obtained as well (Liu et al., 2021). All PBC cases were selected using a self-reported method (Cordell et al., 2015) and the cases of PSC were diagnosed by using standard clinical, biochemical, cholangiographic, and histological criteria (Ji et al., 2017).

The obesity-associated traits include the body mass index (BMI) (Locke et al., 2015), waist-to-hip ratio (WHR) (Shungin et al., 2015), and body fat percentage (BFP) (Lu et al., 2016). Type 2 diabetes mellitus (T2DM) (Mahajan et al., 2014) and its associated indices were included as putative risk factors, namely fasting insulin, fasting glucose, glycated hemoglobin (HbA1c), and 2-h oral glucose tolerance testing (2-h OGTT) (Chen et al., 2021a). Furthermore, other putative risk factors included total cholesterol (TC), triglycerides (TG), low-density lipoprotein cholesterol (LDL-C), high-density lipoprotein cholesterol (HDL-C) (Willer et al., 2013), systolic blood pressure (SBP), and diastolic blood pressure (DBP) (Evangelou et al., 2018). As for smoking and drinking, they were all continuous variables where the average number of cigarettes smoked per day represents smoking and the average number of drinks each week was drinking.

The GWAS of CCA was performed in 635,710 participants, including 476,091 Europeans (832 cases) and 159,619 East Asians (418 cases), adjusting for age, age^2^, sex, age  ×  sex, age^2^   ×  sex, and the first 20 genetic principal components, using SAIGE (v.0.37) to control for the case–control imbalance (Zhou et al., 2018; Sakaue et al., 2021). The genomic control has been applied to each GWAS and details are displayed in Table 1. The IV information can be found in Supplementary Table S1.

**TABLE 1:** Summarized GWAS information of each putative risk factor.

Risk factor	Consortium	Ancestry	Sample size	I^2^	NSNP	R^2^ (%)	F statistic	Covariate	Unit	PMID
Cholelithiasis	Iceland + UKB	European	764,012	0.99	27	0.37	103.95	-	1 unit in logOR	30504769
Cirrhosis	FinnGen	European	218,792	0.96	25	0.37	32.46	age, sex, 10 PCs and genotyping batch	1 unit in logOR	-
PBC	-	European	13,239	0.98	26	13.88	81.87	principal components	1 unit in logOR	26394269
PSC	IPSCSG	Mixed	24,751	0.99	28	19.78	217.65	principal components and genotyping batch	1 unit in logOR	27992413
Chronic pancreatitis	FinnGen	European	196,811	0.94	23	0.35	30.00	age, sex, 10 PCs and genotyping batch	1 unit in logOR	-
NAFLD	GOLD	European	7,176	0.99	5	6.14	93.84	age, age squared, sex, alcohol consumption, and first 10 principal components	1 unit in logOR	21423719
LFC	UKB	European	32,858	0.99	13	5.13	136.70	age at imaging visit, age squared, sex, imaging center, scan date, scan time, and genotyping batch	SD	34128465
HDL-C	GLGC	Mixed	188,577	0.97	125	7.01	113.73	age, age^2^, and sex	SD	24097068
LDL-C	GLGC	Mixed	188,577	0.98	99	7.11	145.74	age, age^2^, and sex	SD	24097068
Total cholesterol	GLGC	Mixed	188,577	0.98	117	7.29	126.65	age, age^2^, and sex	SD	24097068
Triglycerides	GLGC	Mixed	188,577	0.98	71	5.09	142.45	age, age^2^, and sex	SD	24097068
T2DM	DIAGRAM	Mixed	110,452	0.98	26	1.97	85.46	study-specific components	1 unit in logOR	24509480
2-h glucose	MAGIC	European	281,416	0.98	14	0.32	64.67	study-specific covariates	SD	34059833
Fasting glucose	MAGIC	European	281,416	0.99	16	0.73	129.21	study-specific covariates	SD	34059833
Fasting insulin	MAGIC	European	281,416	0.98	43	0.78	51.74	study-specific covariates	SD	34059833
HbA1c	MAGIC	European	281,416	0.99	94	3.16	97.68	study-specific covariates	SD	34059833
SBP	ICBP	European	757,601	0.98	761	6.67	71.04	sex, age, age^2^, BMI and genotyping chip	SD	30224653
DBP	ICBP	European	757,601	0.98	788	7.16	74.03	sex, age, age^2^, BMI and genotyping chip	SD	30224653
IBD	-	Mixed	59,957	0.98	130	16.02	87.76	first ten principal components	1 unit in logOR	28067908
Ulcerative colitis	-	Mixed	45,975	0.99	69	11.41	85.67	first ten principal components	1 unit in logOR	28067908
Crohn’s disease	-	Mixed	40,266	0.99	106	20.53	97.87	first ten principal components	1 unit in logOR	28067908
BMI	GIANT	European	234,069	0.90	95	1.61	40.40	sex, age, age squared, and principal components	SD	25673413
WHR	GIANT	European	210,088	0.92	33	0.46	29.64	age, age square, and study-specific covariates	SD	25673412
BFP	-	European	65,831	0.98	10	0.67	44.60	sex, age, age squared, and study-specific covariates	SD	26833246
Smoking	GSCAN	European	249,752	0.99	28	1.03	92.79	age, sex, age × sex interaction, and the first 10 genetic principle components	SD	30643251
Drinking	GSCAN	European	335,394	0.99	39	0.54	46.37	age, sex, age × sex interaction, and the first 10 genetic principle components	SD	30643251

Notes: GWAS, genome-wide association study; Risk factor, the name of putative risk factors; Consortium, the name of GWAS, consortium; Ancestry, the ethnical background of samples in GWAS; Sample size, the sample size of GWAS; I^2^, the I square to appraise the violation of “No Measurement Error” assumption; NSNP, the number of included single nucleotide polymorphism (SNP); R^2^(%), the proportion of variance in the risk factor explained by SNP; F statistic, the F statistic to appraise weak instrument bias; Covariate, the included covariates of GWAS; Unit, the unit of risk factor; PMID, the publication ID, in the PubMed of GWAS; PBC, primary biliary cholangitis; PSC, primary sclerosing cholangitis; NAFLD, nonalcoholic fatty liver disease; LFC, liver fat content; HDL-C, high-density lipoprotein cholesterol; LDL-C, low-density lipoprotein cholesterol; T2DM, type 2 diabetes mellitus; HbA1c, glycated hemoglobin; SBP, systolic blood pressure; DBP, diastolic blood pressure; IBD, inflammatory bowel disease; BMI, body mass index; WHR, waist-to-hip ratio; BFP, body fat percentage; UKB, United Kingdom Biobank; IPSCSG, International PSC Study Group; GOLD, Genetics of Obesity-related Liver Disease; GLGC, Global Lipids Genetics Consortium; DIAGRAM, DIAbetes Genetics Replication And Meta-analysis consortium; MAGIC, the Meta-Analyses of Glucose and Insulin-related traits Consortium; ICBP, International Consortium of Blood Pressure; GIANT, Genetic Investigation of ANthropometric Traits; GSCAN, GWAS and Sequencing Consortium of Alcohol and Nicotine use; OR, odds ratio; SD, standard deviation.

### Study design and IV selection

**FIGURE 1:**
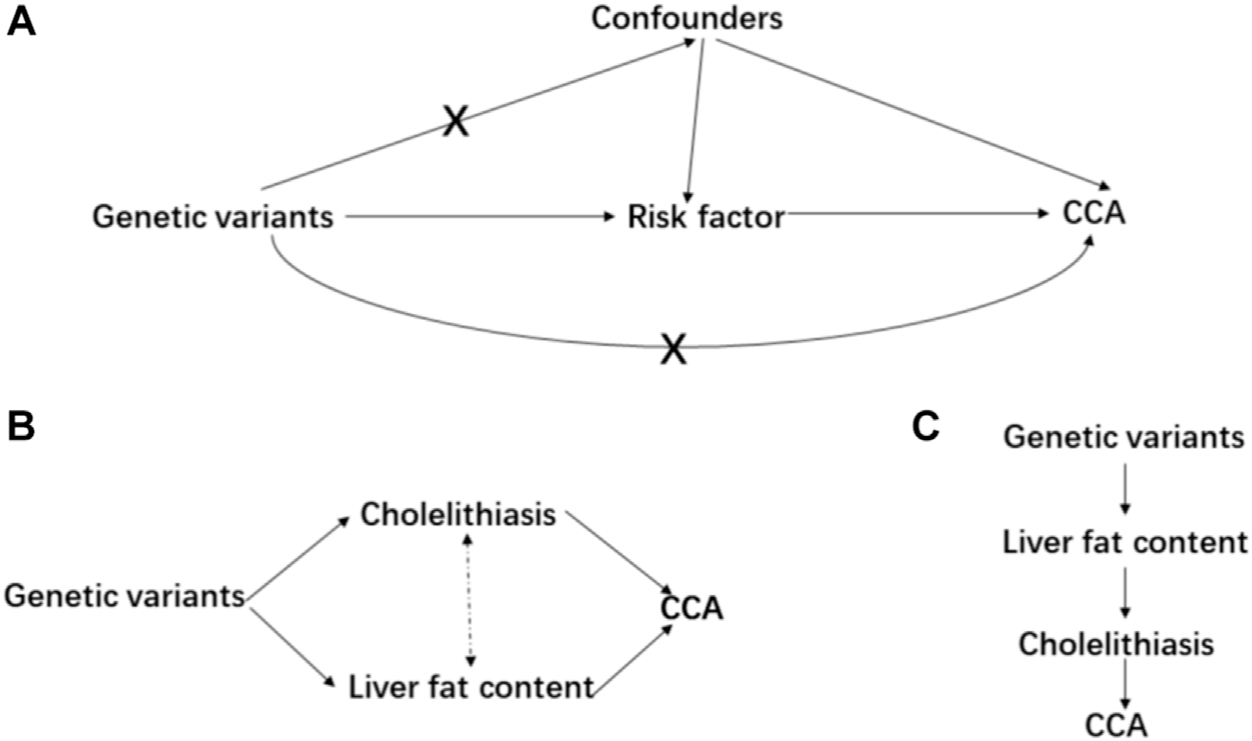
Main design of this study. **(A)** consists of the basic assumptions of the Mendelian randomization; **(B)** is the design of multivariable MR; and **(C)** is the design of mediation MR.

Mendelian randomization is implemented on three principal assumptions (Banales et al., 2020) relevance: the genetic variant, usually single nucleotide polymorphism (SNP), should be closely associated with the exposure; (Banales et al., 2016) independence: the genetic variant should not be associated with any potential confounders that might affect the exposure–outcome association; (Abou-Alfa et al., 2020) exclusion restriction: the genetic variant should not be associated with the outcome except *via* the way of exposure (Emdin et al., 2017) (Figure 1A). Furthermore, linearity and no interaction between exposure and mediators should also be satisfied (Lawlor et al., 2008). Initially, a two-sample MR analysis was applied to estimate the causal effect between each pair of exposure–outcome associations. For significant risk factors derived from the two-sample MR, the multivariable MR analysis was performed to judge whether these causal risk factors are independent of each other (Sanderson et al., 2019) (Figure 1B). Considering cholelithiasis as a direct and strong risk factor, we further hypothesized it to be the mediator and tested this hypothesis using mediation analysis (Carter et al., 2021) (Figure 1C). Preliminarily, SNPs reaching the genome-wide significance (*p*-value < 5 × 10^−8^) were selected as IVs and they were further clumped based on the genomic region (up-/downstream 1 Mb) and linkage disequilibrium (LD, r^2^ = 0.01). If the number of IVs is below 3, a less stringent genome-wide significant threshold would be adopted (*p*-value < 1 × 10^−5^) (liver cirrhosis and chronic pancreatitis, Table 1).

### Statistical methods

For each exposure, the F statistic was calculated to appraise the weak instrument bias (Burgess and Thompson, 2011) (Table 1). Also, to judge whether the genetic variants firstly alter the exposure level and then affect the CCA *via* exposure, the MR Steiger directionality test was performed (Hemani et al., 2017). An inverse-variance weighted method was used as the main method and the fixed-effects model was adopted if there was no heterogeneity. If heterogeneity existed, a multiplicative random-effects model would be used. Then, we used the weighted median estimator (Bowden et al., 2016a), a method that can provide valid estimates even when up to 50% of the included SNPs are invalid instruments (Bowden et al., 2016a), and the MR-Egger regression (Bowden et al., 2015), a method generally considered to be conservative under violations of IV assumptions, was adopted in the presence of horizontal pleiotropy (Slob and Burgess, 2020). In MR-Egger, I square statistic (I_GX_^2^) was used to appraise the violation of the “No Measurement Error” (NOME) assumption (Bowden et al., 2016b) (Table 1). An IVW-based method was used in the multivariable MR analysis (Sanderson et al., 2019) and another regression-based method was adopted as well where we regress the residuals of the outcome, which were obtained from the regression model on the other exposures, on the coefficients of each exposure (Burgess and Thompson, 2015). In the mediation MR analysis, two methods were adopted called the “Product of coefficients method” and “Difference in coefficients method”, and a bootstrap method was used to obtain the 95% confidence interval (Carter et al., 2021).

### Sensitivity analysis

In further sensitivity analyses, the leave-one-out sensitivity analysis and MR-PRESSO methods were used to detect outliers that could distort or drive the main results (Liu et al., 2019). We also performed the weighted mode-based estimation method, which provides the strongest estimates when the most common causal effect estimate is a consistent estimate of the true causal effect, even if the majority of instruments are invalid (Hartwig et al., 2017). The false discovery rate (FDR) was applied to control the false–positive rate in multiple comparisons. The “TwoSampleMR”, “MRPRESSO”, and “MVMR” packages were used in the analytic processes (Verbanck et al., 2018; Walker et al., 2019; Sanderson et al., 2021). Furthermore, we appraised the statistical power (https://cnsgenomics.shinyapps.io/mRnd/) (Brion et al., 2013) and sample overlapping bias (https://sb452.shinyapps.io/overlap/) (Burgess et al., 2016).

## Results

### Brief description of the selected IV

The smallest F statistic of all 26 risk factors was 30, greater than the empirical threshold of 10, and it suggested there should be less weak instrument bias in the analyses (Table 1). The number of included IVs ranged from 5 to 788 and each IV’s F statistic was above 10. The MR Steiger directionality test indicated that all directions are true, meaning the IV estimates are less likely to be biased by reversal causation. The NOME assumption is well satisfied as all I_GX_^2^ statistics are greater than 0.9. All these aforementioned factors guaranteed that the selected IVs are valid.

### Main results of two-sample MR

Mainly, three risk factors are significant after FDR correction, namely LFC, cholelithiasis, and NAFLD. Furthermore, LDL-C and TC are marginally, causally associated with CCA where a marginal causal association was defined to be the original *p*-value < 0.1 while failing to pass the FDR correction.

The odds of CCA would increase with per 1-SD increase in genetically-determined LFC (OR = 2.12 [1.66, 2.71], *p*-value = 1.43 × 10^−9^). The genetic predisposition to cholelithiasis and NAFLD should also elevate the risk of CCA with per unit increase in logOR (cholelithiasis: OR = 2.17 [1.47, 3.20], *p*-value = 9.17 × 10^−5^; NAFLD: OR = 1.53 [1.18, 1.97], *p*-value = 0.001). Two marginally associated risk factors are LDL-C (OR = 0.84 [0.71, 0.99], *p*-value = 0.040) and TC (OR = 0.84 [0.71, 1.01], *p*-value = 0.060); however, genetically elevated LDL-C and TC might decrease the risk of CCA.

**FIGURE 2:**
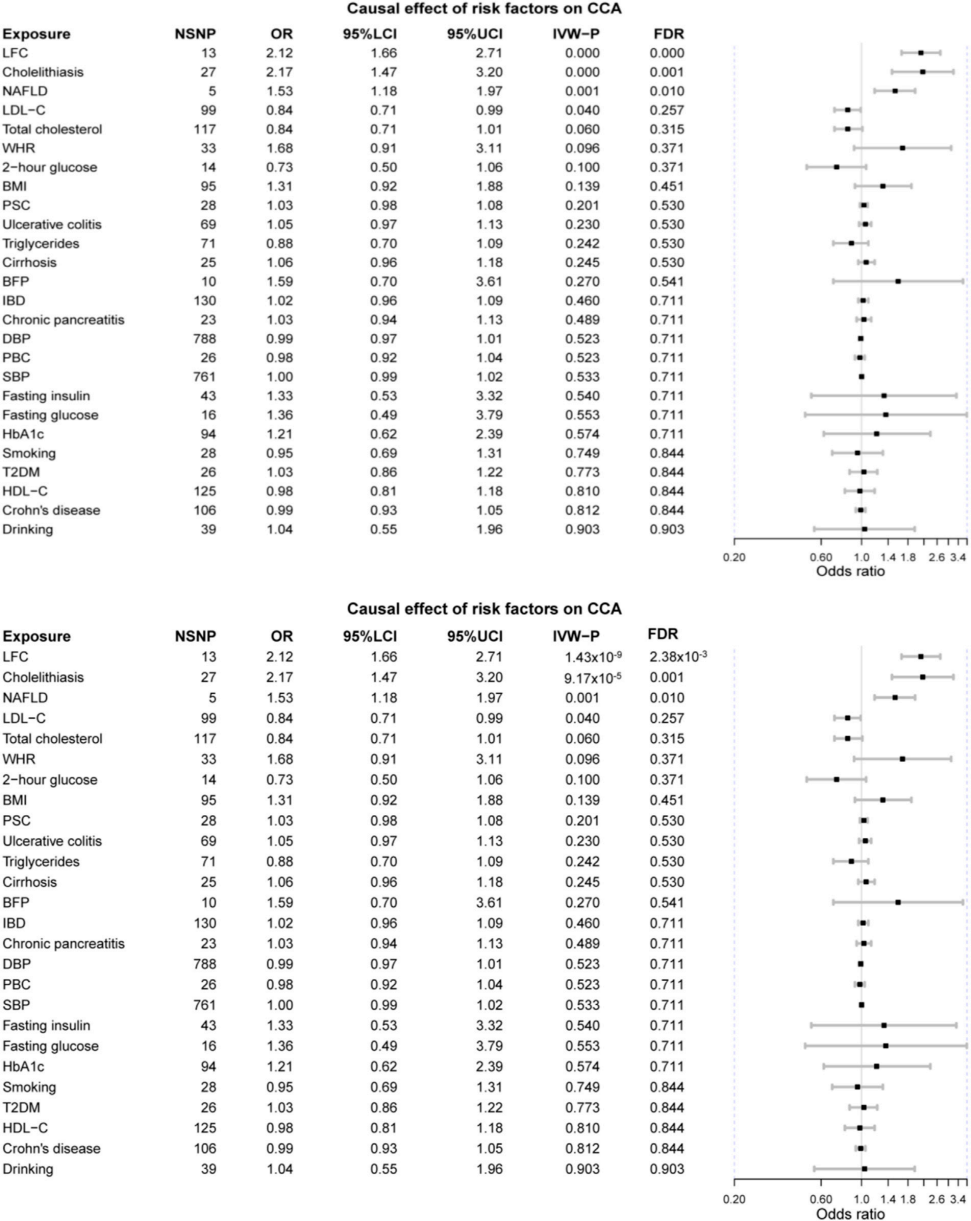
The forest plot of MR results. LFC, liver fat content; NAFLD, non-alcoholic fatty liver disease; LDL-C, low-density lipoprotein cholesterol; WHR, waist-to-hip ratio; BMI, body mass index; PSC, primary sclerosing cholangitis; BFP, body fat percentage; IBD, inflammatory bowel disease; DBP, diastolic blood pressure; PBC, primary biliary cholangitis; SBP, systolic blood pressure; HbA1c, glycated hemoglobin; T2DM, type 2 diabetes mellitus; HDL-C, high-density lipoprotein cholesterol.

It should be noted that other metabolic exposures were not causally associated with CCA, including obesity-related indices (WHR: OR = 1.68 [0.91, 3.11], *p*-value = 0.096; BMI: OR = 1.31 [0.92, 1.88], *p*-value = 0.139; BFP: OR = 1.59 [0.70, 3.61], *p*-value = 0.270), T2DM (OR = 1.03 [0.86, 1.22], *p*-value = 0.773), and blood pressure (DBP: 0.99 [0.97, 1.01], *p*-value = 0.523; SBP: OR = 1.00 [0.99, 1.02], *p*-value = 0.533). Furthermore, no obvious causal association was detected between the other putative risk factors and CCA, even for those factors in strong association with CCA in the previous meta-analysis, such as PSC (OR = 1.03 [0.98, 1.08], *p*-value = 0.201) and liver cirrhosis (OR = 1.06 [0.96, 1.18], *p*-value = 0.245) (Figure 2).

The MR-PRESSO method detected outliers that might distort the MR results for LFC, cholelithiasis, and NAFLD, and the causal estimates were still significant after the removal of these outliers (LFC OR = 1.91 [1.28, 2.84], *p*-value = 0.009; cholelithiasis OR = 1.82 [1.17, 2.84], *p*-value = 0.014; NAFLD = 1.91 [1.33, 2.75], *p*-value = 0.039). The MR-Egger intercepts of them were not different from zero, suggesting there might be no horizontal pleiotropy in the MR analyses. Additionally, there was no heterogeneity after the removal of the outliers detected by MR-PRESSO. The MR estimates of these three risk factors were significant in weighted-median and weighted-mode methods (Table 2). No outliers were detected for LDL-C and TC. Either heterogeneity or horizontal pleiotropy was found for TC while a slight heterogeneity was found in LDL-C (Table 2). The statistical powers of LFC and NAFLD were 100% and that of cholelithiasis was 75%, suggesting sufficient statistical power in our study. Considering the proportion of overlapped samples should be less than 30% and its type 1 error rate is less than 0.05 suggests that our MR results were robust.

**TABLE 2:** Mendelian randomization results of the other applied methods.

Exposure	IVW-MRE					MR-Egger				Weighted median				Weighted mode					
	NSNP	OR	95% LCI	95% UCI	*p*	OR	95% LCI	95% UCI	*p*	OR	95% LCI	95% UCI	*p*	OR	95% LCI	95% UCI	p	P_heterogeneity_	P_pleiotropy_
2-h glucose	14	0.73	0.50	1.05	0.088	0.47	0.18	1.27	0.165	0.66	0.39	1.10	0.110	0.63	0.35	1.16	0.163	0.518	0.376
BFP	10	1.59	0.46	5.49	0.465	3.05	0.01	1224.44	0.724	1.70	0.50	5.77	0.395	2.01	0.34	11.81	0.458	0.015	0.832
BMI	95	1.31	0.90	1.92	0.162	0.89	0.35	2.26	0.804	1.22	0.64	2.34	0.549	1.33	0.56	3.16	0.522	0.199	0.372
Cholelithiasis	27	2.17	1.35	3.49	0.001	2.22	0.96	5.12	0.073	2.07	1.14	3.74	0.016	1.95	1.12	3.40	0.025	0.053	0.950
Chronic pancreatitis	23	1.03	0.95	1.12	0.418	1.08	0.92	1.26	0.342	1.11	0.97	1.28	0.143	1.09	0.92	1.29	0.316	0.814	0.493
Cirrhosis	25	1.06	0.92	1.23	0.404	1.28	0.96	1.70	0.105	1.01	0.86	1.19	0.860	0.99	0.75	1.30	0.945	0.004	0.159
Crohn’s disease	106	0.99	0.93	1.06	0.818	0.94	0.79	1.11	0.466	0.98	0.90	1.08	0.695	1.02	0.88	1.19	0.783	0.279	0.488
DBP	788	0.99	0.97	1.01	0.519	0.99	0.94	1.05	0.782	1.00	0.97	1.03	0.998	1.03	0.93	1.13	0.620	0.649	0.977
Drinking	39	1.04	0.53	2.04	0.910	1.19	0.48	2.96	0.716	1.23	0.56	2.68	0.608	1.18	0.56	2.52	0.665	0.251	0.673
Fasting glucose	16	1.36	0.36	5.18	0.650	1.34	0.11	16.55	0.823	1.52	0.43	5.32	0.514	1.31	0.33	5.19	0.703	0.043	0.987
Fasting insulin	43	1.33	0.50	3.51	0.563	1.91	0.10	35.44	0.666	1.59	0.43	5.91	0.490	1.03	0.10	10.56	0.981	0.266	0.798
HbA1c	94	1.21	0.60	2.47	0.592	0.45	0.11	1.78	0.256	0.88	0.31	2.49	0.808	0.79	0.25	2.49	0.687	0.236	0.103
HDL-C	125	0.98	0.81	1.19	0.816	1.13	0.78	1.62	0.523	0.94	0.70	1.27	0.696	1.11	0.77	1.58	0.581	0.292	0.367
IBD	130	1.02	0.96	1.09	0.466	1.05	0.94	1.17	0.404	0.99	0.87	1.12	0.848	1.02	0.91	1.15	0.678	0.411	0.609
LDL-C	99	0.84	0.68	1.02	0.083	0.82	0.60	1.12	0.209	0.85	0.63	1.13	0.255	0.87	0.66	1.15	0.331	0.005	0.859
LFC	13	2.12	1.34	3.36	0.001	3.15	1.57	6.31	0.008	2.10	1.55	2.85	<0.001	2.08	1.52	2.86	0.001	<0.001	0.177
NAFLD	5	1.53	0.93	2.51	0.095	1.93	0.68	5.45	0.302	1.56	1.11	2.19	0.010	2.06	1.40	3.03	0.021	0.005	0.637
PBC	26	0.98	0.92	1.04	0.511	0.94	0.77	1.16	0.594	1.00	0.92	1.09	0.999	0.98	0.87	1.10	0.692	0.547	0.721
PSC	28	1.03	0.98	1.09	0.267	1.02	0.93	1.11	0.727	1.02	0.96	1.09	0.536	1.02	0.96	1.09	0.438	0.121	0.691
SBP	761	1.00	0.99	1.02	0.539	1.01	0.98	1.05	0.405	1.00	0.98	1.02	0.891	1.01	0.97	1.06	0.577	0.282	0.519
Smoking	28	0.95	0.62	1.45	0.809	0.62	0.29	1.33	0.229	0.82	0.50	1.33	0.423	0.82	0.51	1.31	0.412	0.010	0.201
T2DM	26	1.03	0.85	1.23	0.789	1.81	0.97	3.39	0.075	1.11	0.86	1.42	0.439	1.29	0.91	1.82	0.170	0.259	0.076
Total cholesterol	117	0.84	0.70	1.01	0.071	0.76	0.56	1.03	0.081	0.83	0.59	1.15	0.263	0.77	0.55	1.08	0.128	0.257	0.401
Triglycerides	71	0.88	0.68	1.13	0.315	0.59	0.40	0.87	0.009	0.85	0.60	1.21	0.367	0.71	0.50	0.99	0.050	0.026	0.011
Ulcerative colitis	69	1.05	0.97	1.13	0.215	1.07	0.89	1.28	0.500	1.00	0.90	1.12	0.971	0.97	0.79	1.17	0.725	0.624	0.834
WHR	33	1.68	0.76	3.72	0.199	7.24	0.34	154.14	0.214	1.67	0.63	4.40	0.303	2.06	0.43	9.82	0.370	0.009	0.340

Notes: IVW-MRE, inverse variance weighted-multiplicative random effects; NSNP, the number of single nucleotide polymorphism used in the analysis; OR, the odds ratio; 95% LCI, the lower limit of 95% confidence interval; 95% UCI, the upper limit of 95% confidence interval; *p*, the *p*-value of OR; P_heterogeneity_, the *p*-value of heterogeneity test; P_pleiotropy_, the *p*-value of horizontal pleiotropy test; PBC, primary biliary cholangitis; PSC, primary sclerosing cholangitis; NAFLD, nonalcoholic fatty liver disease; LFC, liver fat content; HDL-C, high-density lipoprotein cholesterol; LDL-C, low-density lipoprotein cholesterol; T2DM, type 2 diabetes mellitus; HbA1c, glycated hemoglobin; SBP, systolic blood pressure; DBP, diastolic blood pressure; IBD, inflammatory bowel disease; BMI, body mass index; WHR, waist-to-hip ratio; BFP, body fat percentage.

### Main results of the multivariable MR and mediation analyses

Since NAFLD and LFC are extremely similar and LFC is a continuous variable, we would only include LFC and cholelithiasis in the multivariable and mediation analyses (Carter et al., 2021). In the multivariable MR analysis, the F statistics of LFC and cholelithiasis were 29.13 and 67.49, respectively. The multivariable MR suggested cholelithiasis (OR = 1.16 [1.01, 1.34], *p*-value = 0.043) and LFC (OR = 1.88 [1.39, 2.55], *p*-value = 1.53 × 10^−4^). However, the Q-statistic for instrument validity was 80.26 (*p*-value = 0.009), suggesting the original estimates can be susceptible to weak instruments caused by a pleiotropic bias. In another complementary regression analysis based on residuals, the previously observed significant results still held while cholelithiasis (OR = 1.26 [1.13, 1.41], *p*-value = 2.66 × 10^−5^) appeared more significant than LFC (OR = 1.71 [1.22, 2.39], *p*-value = 0.002). The two different analytic methods suggested that LFC and cholelithiasis are two independent risk factors for CCA.

The mediation MR analyses consisted of two methods, namely the “product of coefficients method” and the “difference in coefficients method”. The results reached convergent with iterations of bootstrap over 3,000. Therein, the indirect effect size of LFC on CCA that might be mediated by cholelithiasis was not significant in the “product of coefficients method” (OR = 0.95 [0.85, 1.06], *p*-value = 0.806). Also, the results of the indirect effect remained insignificant in “difference in coefficients method” (OR = 0.95 [0.85, 1.07], *p*-value = 0.828).

The results of multivariable and mediation MR analyses suggested a genetic predisposition to higher LFC and cholelithiasis are two independent risk factors of CCA and the effect of LFC on CCA might not be mediated by cholelithiasis.

## Discussion

Our Mendelian randomization systematically examined the causal relationship between previously-established risk factors and CCA. The results suggested that the genetic predispositions to higher LFC, NAFLD, and cholelithiasis were causal risk factors of CCA, and LFC and cholelithiasis were two independent ones. The genetically-lowered serum TC and LDL-C might increase the risk of CCA marginally. Furthermore, it should be noted that some strong risk factors derived from the observational studies were not significant in our study, especially for liver cirrhosis and PSC.

An American study suggested that NAFLD was associated with CCAs (Petrick et al., 2017), which is also confirmed in a recent Korean cohort study demonstrating that the adjusted hazard ratio was 1.33 (Park et al., 2021a), relatively smaller than the MR estimate (MR OR = 1.53). Although there is a lack of direct evidence pointing to the association between LFC and CCA from previous studies, it is reasonable to consider LFC and NAFLD together since the LFC was quantified by the magnetic resonance imaging-derived proton density fat fraction (MRI-PDFF) in UKB and MRI-PDFF has been a recommended indicator for the NAFLD diagnosis (Caussy et al., 2018). As CCA was often initiated by biliary inflammation and cholestasis and it can be categorized into “inflammation” and “proliferation” based on the transcriptomic profile, NAFLD may elevate the risk of CCA by causing the inflammation response *via* the IL6-STAT3 signaling pathway and promoting cell growth *via* the receptor tyrosine kinase (RTK) signaling pathway (Wieckowska et al., 2008; Michelotti et al., 2013; Banales et al., 2020). Chronic inflammation caused by NAFLD can elevate the DNA mutation rate *via* reactive oxygen stress and DNA damage, and damaged biliary epithelial cells, thereby contributing to tumorigenesis (Yang et al., 2019). Recently, researchers used a gene knockout mice model to simulate cholangitis-associated liver cancer where after being fed with a high-fat diet, the mice developed severer cholangitis as well as an increased number of HCC and CCA, indicating a causal relationship between NAFLD and CCA (Maeda et al., 2021). However, the mechanisms involved in NAFLD promoting extrahepatic CCA (eCCA) remain unclear.

Another established risk factor in our study is cholelithiasis, and it also has been identified as a prominent CCA risk factor, especially for eCCA (extrahepatic CCA) in many studies (Cai et al., 2015; Clements et al., 2020; Barner-Rasmussen et al., 2021) and a possible explanation for its CCA-promoting mechanism should be the continuous chronic inflammation of bile duct caused by gallstones (Brindley et al., 2021). Furthermore, its risk effect on CCA might decline with time after cholecystectomy (Nordenstedt et al., 2012), which equals to removing the chronic inflammatory stimulus. Intriguingly, the effect of cholelithiasis on CCA is independent of that of LFC and it does not mediate the effect of LFC on CCA as well, suggesting that cholelithiasis and LFC can affect CCA *via* distinct mechanisms and the common chronic inflammation process cannot explain it. There was little overlap between NAFLD-associated and cholelithiasis-associated SNPs, suggesting the substantial difference in genetic determinants between NAFLD and cholelithiasis might help account for the independent effects. Further research should be carried out to clarify it. Additionally, cholelithiasis can elevate the risk of chronic pancreatitis, and the observed association of chronic pancreatitis with CCA can be biased by cholelithiasis as no direct causation was detected in this MR study (Yuan et al., 2021).

However, we did not observe the casual effect of PSC on CCA, which was in contrast to the previously observed results. The reported prevalence of CCA was estimated to be 7% while that of cholelithiasis was approximately 25% in PSC patients (Karlsen et al., 2017), suggesting that a previously observed association between PSC and CCA can be confounded by cholelithiasis, a confirmed causal risk factor in this study. Also, the incidence of CCA should vary among different types of PSC patients where those with dominant strictures are at the highest risk (up to 76% being perihilar), whereas small duct PSC patients are at a lower risk (Dyson et al., 2018). However, the used GWAS did not categorize PSC and CCA, and the derived null results might be dominated by the null association between specific types. Further investigations should consider cholelithiasis when exploring the relationship between PSC and CCA, and a detailed stratification of PSC and CCA is encouraged as well.

IBD has been associated with CCA while such an association was not confirmed in this MR study. Such results can be explained by the subtypes of CCA and the existing confounders as well. Liver cirrhosis is considered a decisive factor of CCA, especially for iCCA (Razumilava and Gores, 2014). However, we did not observe such a causation and at least two reasons can account for it (Banales et al., 2020). The NAFLD-drive CCA might be a driver, mechanistically distinct from cirrhosis-driven CCA and the observed association between cirrhosis and CCA should be biased by NAFLD (Foerster et al., 2021; Banales et al., 2016); the liver cirrhosis can mainly promote iCCA but not for eCCA and the MR estimate was null since the CCA GWAS encompassed both iCCA and eCCA (Abou-Alfa et al., 2020); the liver cirrhosis is a complex disease caused by different risk factors like HBV and HCV while there is a lack of stratified GWAS for cirrhosis based on risk factors. Thus, it is a necessity to perform GWAS for iCCA and eCCA separately, together with a stratified GWAS for liver cirrhosis, and the evidence would be more convincing with a sophisticated stratification.

Whether obesity can elevate the risk of CCA is still inconsistent and the current evidence appears to endorse a more established association with Europeans than Asians (Osataphan et al., 2021). Our MR analysis supported that there was no direct causation between obesity and CCA since none of the obesity-related indices can contribute to the CCA risk (BMI, WHR, and BFP). Considering that obesity can alter levels of adipokines, pro-tumorigenic lipids, and metabolites, and since lipid accumulation is an established causal risk factor in our study, it might be reasonable that the previously observed association between obesity and CCA, especially for iCCA, could be mediated by LFC (Osataphan et al., 2021). The circumstances of T2DM and its associated indices are similar to that of obesity where the MR analysis suggested a null association. At the same time, most of the observational studies reported that T2DM and impaired fasting glucose were associated with increased risk of both iCCA and eCCA (Clements et al., 2020; Petrick et al., 2018; Park et al., 2021b). Like obesity, the crosstalk in T2DM and NAFLD should help to explicate such circumstances since NAFLD can promote both diabetes and obesity (Liu et al., 2020). The largest meta-analysis did not find an association between hypertension and CCA and our MR analysis supported it. Furthermore, these MR analyses displayed that genetically-elevated blood LDL-C and TC could reduce the risk of CCA marginally, which might be explained by the risk-lowering effect of genetically elevated blood LDL-C and TC on cholelithiasis (Chen et al., 2021b).

The largest meta-analysis indicated that both smoking and drinking were risk factors for CCA (5) while our results suggested that such associations should not be directly causal. Previous studies suggested that smoking can increase the risk of NAFLD and cholelithiasis and it is no surprise that there existed an association between smoking and CCA (Chen et al., 2021b; Jung et al., 2019). Additionally, it has been well established that alcohol intake can damage the liver and cause lipid accumulation. Thus, there might be no direct causal link between alcohol intake and CCA.

This MR study fills the gap of the empirical risk factors of CCA between basic research and clinical observation to some extent. It has several strengths as follows: (Banales et al., 2020): the large sample size of CCA GWAS; (Banales et al., 2016); the MR design can help to infer causation using observational data; (Abou-Alfa et al., 2020); the utility of genetic variants can help free this study from undetectable confounders. However, the limitations should be pointed out as well (Banales et al., 2020). Horizontal pleiotropy is a natural flaw of Mendelian and it can distort the MR estimates despite the application of various methods to minimize it (Banales et al., 2016); most of the included samples are Europeans and the generalizability of our conclusions should be limited (Abou-Alfa et al., 2020); the subtypes of CCA were not taken into consideration due to the unavailability of individual-level data (Zhu et al., 2021); the exposures in the MR analysis are genetically determined and are not equal to the observed exposures in epidemiological studies, and MR results cannot overwhelm the traditional epidemiological studies. Anyway, our MR study gave novel insights into the causal risk factors of CCA where NAFLD can increase the risk of CCA independent of cholelithiasis.

Briefly, this MR study confirmed two causal risk factors of CCA, including NAFLD (LFC) and cholelithiasis. However, other established risk factors have not been confirmed. Mainly, three reasons can help to explain it (Banales et al., 2020): previous observed associations between unconfirmed risk factors and CCA should be confounded or mediated by cholelithiasis/NAFLD (Banales et al., 2016); the unconfirmed risk factors should have an impact on the different subtypes of CCA and the casual estimates might attenuate to 0 if analyzing CCA without stratification. Considering the latter, we cannot eradicate the potential causal relationship between these unconfirmed risk factors and CCA, especially for PSC. Furthermore, a more sophisticated and well-elaborated clinical design is recommended to clarify the unsettled questions with an enlarged sample size where the GWAS focused on stratified CCA (iCCA and eCCA) should be carried out since the two types should have different biological mechanisms according to previous literature. Due to the relatively small sample size for both types, we had to combine them to get enough statistical power in this study. However, with an enlarged sample size and CCA stratification, we might be able to gain deep insights into the pathogenesis of CCA. Furthermore, we would explore the independent mechanisms of NAFLD-associated CCA and cholelithiasis-associated CCA and find potential therapeutics for them.

## Conclusion

This MR study examined the causal relationship between putative risk factors and CCA, confirming the hazardous effect of NAFLD and cholelithiasis on it. Our study provides promising modifiable ways of preventing CCA.

## Data Availability

Publicly available datasets were analyzed in this study. These data can be found here: https://www.ebi.ac.uk/gwas/.

## Glossary

CCA: cholangiocarcinoma
MR: Mendelian randomization
GWAS: genome-wide association study
IVW: inverse-variance weighted
LFC: liver fat content
NAFLD: nonalcoholic fatty liver disease
FGFR: fibroblast growth factor receptor
IDH: isocitrate dehydrogenase
PSC: primary sclerosing cholangitis
T2DM: type 2 diabetes mellitus
IBD: inflammatory bowel disease
IV: instrumental variable
RCT: randomized control trial
GBC: gallbladder cancer
PBC: primary biliary cholangitis
UKB: United Kingdom Biobank
ICD: international classification of diseases
GOLD: the Genetics of Obesity-Related Liver Disease
BMI: body mass index
WHR: waist-to-hip ratio
BFP: body fat percentage
HbAa1c: glycated hemoglobin
OGTT: oral glucose tolerance testing
GLGC: the Global Lipids Genetics Consortium
TC: total cholesterol
TG: triglycerides
LDL-C: low-density lipoprotein cholesterol
HDL-C: high-density lipoprotein cholesterol (HDL-C)
SBP: systolic blood pressure; diastolic blood pressure
SNP: single nucleotide polymorphism
NOME: No Measurement Error
FDR: false discovery rate
MRI-PDFF: magnetic resonance imaging-derived proton density fat fraction
eCCA: extrahepatic cholangiocarcinoma
iCCA: intrahepatic cholangiocarcinoma
